# Correction: Dueñas et al. Assessing Effectiveness of Colonic and Gynecological Risk Reducing Surgery in Lynch Syndrome Individuals. *Cancers* 2020, *12*, 3419

**DOI:** 10.3390/cancers13133104

**Published:** 2021-06-22

**Authors:** Nuria Dueñas, Matilde Navarro, Àlex Teulé, Ares Solanes, Mònica Salinas, Sílvia Iglesias, Elisabet Munté, Jordi Ponce, Jordi Guardiola, Esther Kreisler, Elvira Carballas, Marta Cuadrado, Xavier Matias-Guiu, Napoleón de la Ossa, Joan Lop, Conxi Lázaro, Gabriel Capellá, Marta Pineda, Joan Brunet

**Affiliations:** 1 Hereditary Cancer Program, Catalan Institute of Oncology-IDIBELL, ONCOBELL, Hospitalet de Llobregat, 08908 Barcelona, Spain; nduenas@iconcologia.net (N.D.); mnavarrogarcia@iconcologia.net (M.N.); ateule@iconcologia.net (À.T.); msalinas@iconcologia.net (M.S.); siglesias@iconcologia.net (S.I.); emunter@iconcologia.net (E.M.); clazaro@iconcologia.net (C.L.); gcapella@iconcologia.net (G.C.); mpineda@iconcologia.net (M.P.); 2 Centro de Investigación Biomédica en Red de Cáncer (CIBERONC), Instituto Salud Carlos III, 28029 Madrid, Spain; 3 Hereditary Cancer Program, Catalan Institute of Oncology, 08916 Badalona, Barcelona, Spain; asolanes@iconcologia.net; 4 Department of Gynecology, Bellvitge University Hospital, Hospitalet de Llobregat, 08908 Barcelona, Spain; jponce@bellvitgehospital.cat; 5 Department of Gastroenterology, Bellvitge University Hospital, Hospitalet de Llobregat, 08908 Barcelona, Spain; jguardiola@bellvitgehospital.cat; 6 Department of General Surgery, Bellvitge University Hospital, Hospitalet de Llobregat, 08908 Barcelona, Spain; ekreisler@bellvitgehospital.cat; 7 Department of Gynecology, Trias i Pujol University Hospital, 08916 Badalona, Barcelona, Spain; ecarballas.germanstrias@gencat.cat; 8 Department of General Surgery, Trias i Pujol University Hospital, 08916 Badalona, Barcelona, Spain; cuadradin@gmail.com; 9 Department of Pathology, Bellvitge University Hospital, Hospitalet de Llobregat, 08908 Barcelona, Spain; fjmatiasguiu.lleida.ics@gencat.cat; 10 Department of Pathology, Trias i Pujol University Hospital, 08916 Badalona, Barcelona, Spain; napoleondelaossa@gmail.com; 11 Department of Pathology, Hospital General de Catalunya—Grupo Quironsalud, 08203 Barcelona, Spain; 12 Department of Pathology, Hospital del Mar Institute for Medical Research, 08003 Barcelona, Spain; lopgros@gmail.com; 13 Hereditary Cancer Program, Catalan Institute of Oncology-IDBIGI, 17007 Girona, Spain

In the original article, there was a mistake in Figure 3 as published [1]. The image presented corresponded to endometrial cancer-specific mortality cumulative incidence, instead of the figure referred to in the title (all-cause mortality cumulative incidence).

The corrected Figure 3 appears below:

The study carried out is an analysis of cumulative incidence of mortality and not an analysis of overall survival, as explicitly reported in the methodology section (Sections 4.3 and 4.4). Our study, like other retrospective studies could suffer from bias. For that reason, we did a balanced discussion of the strengths and weaknesses of our work in the Discussion section (paragraphs 11, 13 and 14) discussing limitations of retrospective studies, possible cohort effects, selection of patients based on clinical characteristics, etc.). Moreover, to facilitate the reader’s understanding of the text, we would like to refine the conclusions of our work as follows, making them more precise: 

“In conclusion, this study confirms that colonic and gynecological risk reducing surgeries are effective at decreasing the incidence of metachronous colorectal and gynecological cancer in Lynch syndrome (LS) patients. This benefit was seen in all LS subjects; however, caution is still needed for *MSH6* and *PMS2* pathogenic variant carriers. Also, our results point to a reduction in the endometrial and ovarian cancer-specific mortality cumulative incidence in females with LS that undergo risk reducing gynecological surgery. Differences in all-cause mortality cumulative incidence should be confirmed in prospective analyses.” 

The authors apologize for any inconvenience. All the authors have checked and agreed with the corrected paper content. The original article has been updated.

## Figures and Tables

**Figure 3:**
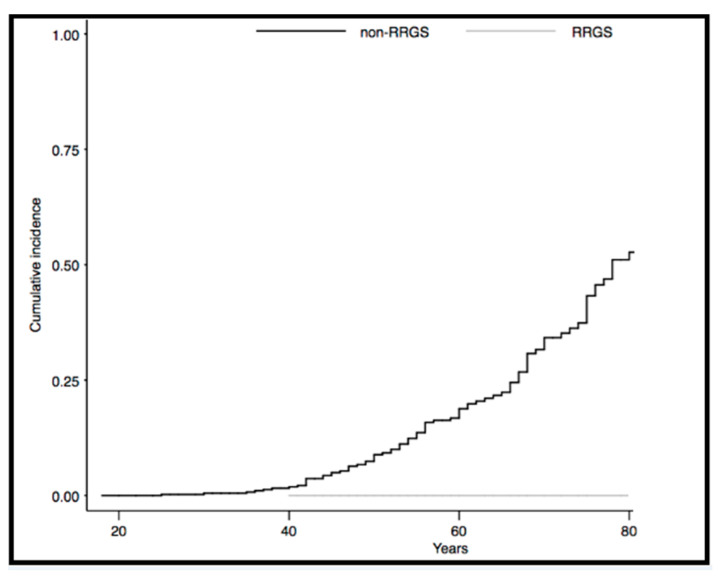
All-cause mortality cumulative incidence in females with Lynch syndrome comparing risk reducing gynecological surgery and non-risk reducing gynecological surgery: All-cause mortality cumulative incidence was 0.0% for risk reducing gynecological surgery vs. 52.7% for non-risk reducing gynecological surgery (*p* = not assessable).
