# Supplementary material for: Assessing Effectiveness of Colonic and Gynecological Risk Reducing Surgery in Lynch Syndrome Individuals
Source: Cancers (Basel). 2020 Nov 18;12(11):3419. doi: 10.3390/cancers12113419 (PMC7698735; doi:10.3390/cancers12113419)
Supplement: Supplementary file 1 [file cancers-12-03419-s001.pdf]

cancers

MDPI

Article

Assessing Effectiveness of Colonic and Gynecological Risk Reducing Surgery in Lynch Syndrome Individuals

Nuria Dueñas [1,2,†], Matilde Navarro [1,2,3,†], Àlex Teulé [1], Ares Solanes [3], Mònica Salinas [1,2], Sílvia Iglesias [1,2], Elisabet Munté [1], Jordi Ponce [4], Jordi Guardiola [5], Esther Kreisler [6], Elvira Carballas [7], Marta Cuadrado [8], Xavier Matias-Guiu [9], Napoleón de la Ossa [10,11], Joan Lop [12], Conxi Lázaro [1,2], Gabriel Capellá [1,2], Marta Pineda [1,2,‡] and Joan Brunet [1,2,13,*,‡]

1. Hereditary Cancer Program, Catalan Institute of Oncology-IDIBELL, ONCOBELL, Hospitalet de Llobregat, 08908 Barcelona, Spain; nduenas@iconcologia.net (N.D.); mnavarrogarcia@iconcologia.net (M.N.); ateule@iconcologia.net (A.T.); msalinas@iconcologia.net (M.S.); siglesias@iconcologia.net (S.I.); emunter@iconcologia.net (E.M.); clazaro@iconcologia.net (C.L.); gcapella@iconcologia.net (G.C.); mpineda@iconcologia.net (M.P.), jbrunet@iconcologia.net (J.B.)
2. Centro de Investigación Biomédica en Red de Cáncer (CIBERONC), Instituto Salud Carlos III, 28029 Madrid, Spain; nduenas@iconcologia.net (N.D.); mnavarrogarcia@iconcologia.net (M.N.); msalinas@iconcologia.net (M.S.); siglesias@iconcologia.net (S.I.); clazaro@iconcologia.net (C.L.); gcapella@iconcologia.net (G.C.); mpineda@iconcologia.net (M.P.), jbrunet@iconcologia.net (J.B.)
3. Hereditary Cancer Program, Catalan Institute of Oncology, Badalona, 089016 Barcelona, Spain; mnavarrogarcia@iconcologia.net (M.N.); asolanes@iconcologia.net (A.S.)
4. Department of Gynecology, Bellvitge University Hospital, 08908 Hospitalet de Llobregat, 089016 Barcelona, Spain; jponce@bellvitgehospital.cat (J.P.)
5. Department of Gastroenterology, Bellvitge University Hospital, Hospitalet de Llobregat, 08908 Barcelona, Spain; jguardiola@bellvitgehospital.cat (J.G.)
6. Department of General Surgery, Bellvitge University Hospital, Hospitalet de Llobregat, 08908 Barcelona, Spain; ekreisler@bellvitgehospital.cat (E.K.)
7. Department of Gynecology, Trias i Pujol University Hospital, Badalona, 089016 Barcelona, Spain; ecarballas.germanstrias@gencat.cat (E.C.)
8. Department of General Surgery, Trias i Pujol University Hospital, Badalona, 089016 Barcelona, Spain; cuadradin@gmail.com (M.C.)
9. Department of Pathology, Bellvitge University Hospital, Hospitalet de Llobregat, 08908 Barcelona, Spain; fjmatiasguiu.lleida.ics@gencat.cat (X.M.-G.)
10. Department of Pathology, Trias i Pujol University Hospital, Badalona, 089016 Barcelona, Spain; napoleondelaossa@gmail.com (N.O.)
11. Department of Pathology, Hospital General de Catalunya—Grupo Quironsalud, 08203 Barcelona, Spain; napoleondelaossa@gmail.com (N.O.)
12. Department of Pathology, Hospital del Mar Institute for Medical Research, 08003 Barcelona, Spain; lopgros@gmail.com (J.L.)
13. Hereditary Cancer Program, Catalan Institute of Oncology-IDBIGI, 17007 Girona, Spain; jbrunet@iconcologia.net (J.B.)

* Correspondence: jbrunet@iconcologia.net; Tel.: +34-93-260-7959
† These authors contributed equally to this work.
‡ These authors contributed equally to this work.

Received: 14 October 2020; Accepted: 06 November 2020; Published: date

Supplementary

.

cancers

MDPI

Table 1. Stage of first and second metachronous colorectal cancer comparing segmental to extended surgery in individuals with colorectal cancer.

Colorectal cancer stage by type of surgery	PTis	Stage I	Stage II	Stage III	Stage IV	Unknown
			FIRST COLORECTAL CANCER			
**Extended surgery n=29**	1 (3.4%)	5 (17.2%)	11 (37.9%)	8 (27.6%)	1 (3.4%)	3 (10.3%)
**Segmental surgery n=261**	2 (0.8%)	37 (14.2%)	109 (41.8%)	68 (26.1%)	11 (4.2%)	34 (13.0%)
			SECOND COLORECTAL CANCER			
**Extended surgery n=1**	0 (0%)	1 (100%)	0 (0%)	0 (0%)	0 (0%)	0 (0%)
**Segmental surgery n=62**	2 (3.2%)	19 (30.6%)	20 (32.3%)	11 (17.7%)	1 (1.6%)	9 (14.5%)

For second colorectal cancer, only metachronous cancers were considered. When colorectal cancer was synchronous, the highest stage was considered.

Table 2. Stage of endometrial and ovarian cancer according to type of surgery in all LS women with gynaecological cancer.

Gynecologic cancer stage by type of surgery	PTis	Stage I	Stage II	Stage III	Stage IV	Unknown
			ENDOMETRIAL CANCER			
**RRGS n=6**	3 (50%)	1 (16.7%)	1 (16.7%)	1 (16.7%)	0 (0%)	0 (0%)
**non-RRGS n=117**	0 (0%)	57 (48.7%)	8 (6.8%)	8 (6.8%)	4 (3.4%)	39 (33.3%)
			OVARIAN CANCER			
**RRGS n=0**	0 (0%)	0 (0%)	0 (0%)	0 (0%)	0 (0%)	0 (0%)
**non-RRGS n=36**	0 (0%)	16 (44.4%)	2 (5.5%)	7 (19.4%)	0 (0%)	11 (30.6%)

RRGS Risk reducing gynaecological surgery, *non-RRGS* Non-risk reducing gynaecological surgery.

Table 3. Histology of gynaecological cancers diagnosed in the gynaecological cohort.

Histology of gynaecological cancer	Endometrial cancer n=123	Ovarian cancer n=36

**Endometrioid**	74 (60.2%)	11 (30.6%)
**Clear cell**	6 (4.9%)	9 (25.0%)
**Serous/Papillary serous**	4 (3.3%)	7 (19.4%)
**Adenocarcinoma**	1 (0.8%)	1 (2.8%)
**Other**	7 (5.7%)	1 (2.8%)
**Unknown**	31 (25.2%)	7 (19.4%)

Histology of 150 gynaecological cancers diagnosed. *Other* includes mixed and sarcomatoid histology.
